# Case Report: Primary bile acid synthesis defect: first cases series from Tunisia

**DOI:** 10.3389/fped.2026.1895852

**Published:** 2026-09-09

**Authors:** Mouna Zribi, Safa Khatrouch, Hela Boudabous, Sana Ben Messaoud, Loreto Hierro Llanillo, Sophie Laplanche, Anne Spraul, Amel Ben Chehida, Mohamed Slim Abdelmoula

**Affiliations:** 1 Department of Pediatrics and Metabolic Diseases, La Rabta University Hospital, Tunis, Tunisia; 2 Faculty of Medicine of Tunis, University of Tunis El Manar, Tunis, Tunisia; 3 Research Laboratory LR12SP02 “Inherited Metabolic Diseases: Investigation and Management”, La Rabta University Hospital, Tunis, Tunisia; 4 Pediatric Hepatology Department, University Hospital La Paz, Madrid, Spain; 5 Department of Medical Biology, Paris Saint-Joseph Hospital, Paris, France; 6 Department of Biology, Bicêtre Hospital, Le Kremelin Bicêtre, Paris, France

**Keywords:** bile acid synthesis defects, cholestasis, cholic acid (CA), fat-soluble vitamins, liver dieases, steatorrhea, Tunisia., ursodeoxicholic acid

## Abstract

**Background:**

Primary bile acid synthesis defects (BASD) are autosomal recessive disorders causing cholestatic liver disease and fat-soluble vitamin malabsorption. Despite being treatable with cholic acid (CA), BASD remain underdiagnosed, particularly in resource-limited settings. No data have been published from Tunisia.

**Methods:**

A retrospective study of all patients diagnosed with BASD at La Rabta University Hospital in Tunis, between 2013 and 2024 described clinical presentation, biochemical and genetic findings, treatment and outcomes.

**Results:**

Six male patients from three unrelated families were enrolled. The age at symptom onset ranged from the neonatal period to 8 years, with a diagnostic delay spanning from 1.5 months to 5 years. Three distinct phenotypes were observed: cholestasis (3/6), malabsorption (4/6), and non-cholestatic hepatopathy (1/6). Four patients presented with 3β-hydroxysteroid dehydrogenase deficiency, including three siblings homozygous for a novel *HSD3B7* variant (c.748_760del) and their brother, who remained genetically undiagnosed despite extensive investigation. One patient had Δ^4^-3-oxosteroid 5β-reductase (5β-R) deficiency carrying a known *AKR1D1* variant (c.242A>T). Three patients received CA therapy (6.2–13.4 mg/kg/day), achieving complete clinical and biochemical remission during 5–6 years of follow-up. Conversely, three patients died from liver failure before CA treatment could be initiated.

**Conclusions:**

This first Tunisian small series highlights the phenotypic heterogeneity of BASD, even in the same family, and the efficacy of CA therapy when initiated early. Besides low GGT cholestasis, fat-soluble vitamin malabsorption and non-cholestatic liver phenotype require investigation of serum bile acids as well as bile acids in urine. Improved access to specialized diagnostic tools and orphan drug therapies is urgently needed in resource-limited settings.

## Take home message

This first Tunisian series highlights the clinical heterogeneity of primary bile acid synthesis defects, even in the same family and demonstrates that early diagnosis and timely and optimal cholic acid therapy are lifesaving, particularly in resource-limited settings.

## Introduction

Bile acid synthesis defects (BASDs) encompass primary BASDs, caused by seven currently recognized genetic defects (1–4)—among which 3β-hydroxysteroid dehydrogenase type 7 (3β-HSD) (OMIM #607765) and Δ^4^-3-oxosteroid 5β-reductase (5β-R) deficiencies (OMIM #235555) are the most prevalent (5, 6)—and bile acid (BA) conjugation defects, which comprise two known genetic disorders. The latter is out of the scope of this report.

While conjugation defects typically present with low or normal gamma-glutamyl transferase (GGT) cholestasis alongside markedly elevated serum BA concentrations, primary BASD characteristically exhibit low total serum BA despite active cholestasis (1–4). This phenotype result from the accumulation of hepatotoxic intermediates and the deficiency in normal primary BAs: cholic acid (CA) and chenodeoxycholic acid (1–4).

The clinical spectrum of primary BASDs is remarkably broad. Pruritus is typically absent despite active cholestasis, distinguishing these disorders from most other cholestatic liver diseases. Some patients may present in the neonatal period with acute liver failure, including a severe phenotype mimicking neonatal hemochromatosis. Furthermore, extrahepatic manifestations, particularly progressive neurological disorders, have been reported in specific BASD subtypes and represent an important part of the disease spectrum (7).

Without intervention, BASDs characteristically progress to biliary cirrhosis and end-stage liver failure (ESLF), requiring life-saving liver transplantation (5–7).

The phenotypic variability of BASDs, combined with limited availability of specialized diagnostic testing, contributes to significant under-diagnosis (8, 9). While oral CA therapy has transformed the therapeutic landscape (6, 7, 10–12), access remains limited in low- and middle-income countries.

Published data from North Africa remain scarce (13), and no cases have been reported from Tunisia. Understanding the phenotypic and genetic landscape in diverse populations is essential for improving disease recognition.

Herein, we report the first Tunisian series of BASDs to delineate their phenotypic and genotypic spectrum, evaluate long-term CA efficacy and safety and outline the diagnostic and therapeutic challenges encountered in a resource-constrained setting.

## Methods

We conducted a descriptive study of all BASDs patients diagnosed at the Department of Pediatrics and Metabolic Diseases in La Rabta Hospital in Tunis, Tunisia, between 2013 and 2024.

### Inclusion criteria

Pediatric patients (age < 18 years) were eligible for inclusion if they presented with low- or normal-GGT cholestasis, unexplained hepatopathy, or fat-soluble vitamin (FSV) deficiency not attributable to pancreatic or other digestive diseases, together with either an abnormal urinary bile acid (BA) profile or a pathogenic/likely pathogenic variant in a gene encoding a bile acid synthesis enzyme (or a documented familial diagnosis in a sibling).

Patients were classified as having confirmed BASDs if they possessed definitive genetic confirmation or a pathognomonic abnormal urinary BAs profile. Remaining patients meeting the inclusion criteria were classified as highly suspected BASDs.

### Diagnostic investigations

Baseline work-up included standard liver function tests (LFTs) and fat-soluble vitamin (A, D, E, K) profiling, with upper limits of normal (ULN) based on age and sex-specific intervals (14). Male ULN thresholds were 29.9–38 U/L for ALT and 41.5–68.7 U/L for AST. The GGT ULN ranged from 41.5 to 68.7 U/L for ages 11 months to 16 years, and was higher for younger infants (15). Cholestasis was defined as serum conjugated bilirubin >17 μmol/L (> 1 mg/dL), regardless of total bilirubin levels (16). Pediatric acute liver failure was diagnosed using standardized criteria (17).

Serum total BAs concentrations were quantified (normal reference range: 0–10 μmol/L), values below 10 µmol/L in the presence of active cholestasis were classified as inappropriately low. Urinary BAs profiling was performed using Gas chromatography – mass spectrometry (GC-MS) or liquid chromatography-mass spectrometry (LC-MS) at Paris Saint-Joseph Hospital (Paris, France) or outsourced to Cerba laboratories. Specific patterns of atypical BAs and their intermediates were utilized to identify distinct enzyme deficiencies of BAs metabolism (18, 19).

Based on urinary bile acid (BA) profiles, genetic screening was performed in France or Madrid via Sanger sequencing of the *HSD3B7* or *AKR1D1* coding regions. Family screening involved targeted Sanger sequencing of the specific exon and flanking intronic regions. Variants were classified according to American College of Medical Genetics and Genomics (ACMGG) guidelines (20).

### Data collection and analysis

Data were retrospectively extracted from medical records, through December 2024, including demographics, family history, clinical presentation, biochemical results, imaging findings [Liver ultrasound and elastography (FibroScan®)], genetic results, treatment details and outcomes: survival, evolution of clinical, biochemical and imaging parameters, CA doses, adherence (assessed through direct parental interviews) and side effects. Descriptive statistics (medians, ranges, frequencies) were used.

CA therapy was initiated at standard initial doses (6–8 mg/kg/day) administered orally in two divided daily doses during meals. Dose titration was performed progressively based on clinical response, weight gain, and serum/urinary bile acid monitoring, if available. Fat-soluble vitamins were co-administered until clinical and biochemical resolution of deficiency signs was confirmed.

### Ethical considerations

Patient confidentiality was preserved, and parents provided written informed consent for urinary BA analysis, genetic testing, and publication. Urinary BA analysis was conducted via a Theravia-supported diagnostic program using the anonymized Euris© digital platform (NetReps® 4, Version 4.7.52). Institutional Review Board and Ethics Committee approval was obtained from the Ethics Committee of La Rabta Hospital (Approval No. CERB 23/2026).

## Results

### Demographics and family history

Six male patients from three unrelated Tunisian families were included (Table 1). With the exception of Cases 1 and 5, all patients were both genetically and biochemically confirmed. Family 1 comprised four siblings (Cases 1–4) born to first-cousin parents, its pedigree was notable for two previous infant deaths in earlier generations from an unidentified “liver disease”. Families 2 and 3 each presented with a single affected child (Cases 5 and 6) without reported consanguinity or family history. All families originated from northwestern Tunisia.

**Table 1:** Summary of clinical, biochemical, radiological and outcome data of the six studied patients.

	Variable	Case 1	Case 2	Case 3	Case 4	Case 5	Case 6
Epidemiologic data	Family	Family 1				Family 2	Family 3
	Consanguinity	First cousins				No	No
	Family history (liver disease/infant deaths)	+				−	−
	Personal history	Infant asthma	−	Rickets/hypocalcemia	Rickets/hypocalcemia	−	Prematurity Neonatal CMV infection
	Age at symptom onset	16 months	8 years	4 months	4 months	15 days	Day 1
Baseline clinical data	Hepatomegaly/Splenomegaly	+/+	+/−	+/−	+/−	−/−	+/+
	Jaundice/Pruritus	−/−	+/−	−/−	−/−	+/NE*	+/−
	Steatorrhea	+	−	+	+	−	+
	Deep tendon reflexes	Areflexia (lower limbs) secondary to vitamin E deficiency	Normal	Areflexia (lower limbs) secondary to vitamin E deficiency	Areflexia (lower limbs) secondary to vitamin E deficiency	Normal	Normal
	Epistaxis	−	+	−	−	−	−
	Age at diagnostic suspicion	Post-mortem	13 years	4 years	4 years	2 months	2.5 months
Baseline biological data	GGT (U/L)(Reference range)	28(41.5–68.7)	12(41.5–68.7)	21(41.5–68.7)	10(41.5–68.7)	45(20–155)	56–129(20–155)
	AST ( × ULN)	1.5×	1.5–24×	5×	2×	10–25×	1–9×
	ALT ( × ULN)	2.5×	2–14×	6×	1×	9–27×	9–10×
	Low Prothrombin time (PT)	5%–89%	41.7%–81%	47%–72%	58.4%–85%	32%–65%	54%–74%
	CholestasisBT/BD mg/L	+ (very late)30/10	+64.9/55	−17/5	−27/4	+46,7/11,7	+50,8/47.5
	Hypocalcemia	−	−	+	+	−	−
	Low cholesterol	+	−	+	+	NA	−
	Elevated CK	+	+	NA	NA	NA	NA
Diagnosis criteria	Total Serum BA (Reference range: 0–10 µmol/L)	NA	1.37 µmol/L	NA	NA	<1 µmol/L	1.99 µmol/L
	Urinary BA mass spectrometry	NA	3β-HSD defect	3β-HSD defect	3β-HSD defect	NA	5β-R defect
	Genetic testing	NA	*HSD3B7* homozygous (exon 7: c.748_760del)			NA	*AKR1D1* homozygous c.242A>T (p.Asp81Val)
	BASD type	Likely Type 1	Type 1	Type 1	Type 1	Unclassified (Type 2?)	Type 2
	Child and Pugh classification at treatment start	−	A	A	A	B	A
Management	Treatment	−	UDCA ± DEK	CA ± DEK	CA ± DEK	UDCA + D/K	UDCA→CA + DEK
	Age at treatment start	−	13 years	4 years 1 month	4 years 1 month	2 months	Day 1 → 18 months
	CA dose at initiation (mg/kg/day)	−	−	8.3	8.4	−	6.2
	CA maintenance dose (mg/Kg/day)	−	−	10.7	10.9	−	13.4
	Clinical follow-up duration	4 years 2 months	5.5 years	6 years	6 years	4 months	2 years
	CA ou UDCA therapy duration	−	6 months	5 years 11 months	5 years 11 months	2 months	5 years
	Indication for Liver transplantation	+	+	−	−	−	−
Outcome	Age at last Evaluation or at death	5.5 years	13.5 years	10 years	10 years	6 months	6.5 years
	Fibroscan at follow-up	NA	NA	FO	F0	NA	F0–F1
	Outcome	Death (liver failure)	Death (liver failure)	Favorable	Favorable	Death (liver failure)	Favorable

BASD, BA synthesis defects; CA, cholic acid; UDCA, ursodeoxycholic acid; ADEK, fat-soluble vitamins; GGT, gamma-glutamyl transferase; AST, aspartate aminotransferase; ALT, alanine aminotransferase; ULN, upper limit of normal; PT, prothrombin time; CK, creatine kinase; CMV, cytomegalovirus.

* NE, not evaluated (age < 5 months); NA, not available.

### Diagnosis

Median age at onset was 6 months (range: 1 day–8 years) and median age at diagnosis was 4 years (range: 2 months–13 years), reflecting diagnostic delays.

Physical Examination Findings at Baseline

Detailed physical examination at baseline revealed jaundice in 3 patients, hepatomegaly (firm consistency) in 5 patients and splenomegaly in two patients (Cases 1 and 6). Neurological examination highlighted fat-soluble vitamin deficiency signs: profound lower-limb areflexia was present in three patients (Cases 1, 3, and 4) due to severe vitamin E deficiency, while Case 2 presented with active epistaxis and mucosal bleeding secondary to severe vitamin K-dependent coagulopathy. Musculoskeletal evaluation identified severe hypocalcemic rickets with rosary beads and limb pain in Cases 3 and 4. Nutritional evaluation indicated severe growth impairment in Case 6 (weight-for-age *Z*-score: −2.5 SD), whereas other patients maintained preserved growth metrics despite chronic steatorrhea. Stools were notably fatty in 4 cases.

All patients had cytolysis (1.5–27 × ULN), associated to normal GGT (5/6), conjugated hyperbilirubinemia (4/6), coagulopathy (5/6), hypocalcemia (2/6), and elevated creatine kinase (2/6). Serum BAs were low (3.5–8.5 μmol/L) despite cholestasis (Cases 2, 5, 6). Urinary bile acid analysis showed 3β-hydroxy-Δ^5^-BAs in 3 siblings (Cases 2–4) (3β-HSD deficiency) and 3-oxo-Δ^4^-BAs in Case 6 (5β-R deficiency).

Phenotypes

Three overlapping phenotypes were identified:

- Cholestatic (3/6; Cases 2, 5, 6): Pruritus was absent despite cholestasis. Case 6 initially had elevated GGT (500-915 U/L in neonatal period, reference range: 27–183 U/L), likely due to cytomegalovirus co-infection and drug effects (UDCA and phenobarbital).
- Malabsorption (4/6; Cases 1–4): Chronic steatorrhea was constant, despite normal growth. Case 2 had vitamin K deficiency–related epistaxis. Cases 1, 3 and 4 had vitamin E-related areflexia. Cases 3 and 4 had hypo-calcemic rickets.
- Non-cholestatic hepatopathy (1/6): Case 1 presented at 8 years with isolated hepatomegaly and cytolysis. After a 5-year inconclusive diagnostic odyssey, he died from ESLF.

Diagnostic Reasoning and Differential Diagnosis

Primary BASDs were clinically suspected in patients presenting with unexplained liver dysfunction, steatorrhea, or severe fat-soluble vitamin deficiencies. The combination of severe cholestasis with low or normal serum GGT levels and paradoxically low total serum bile acids served as the primary biochemical signature to differentiate BASDs from progressive familial intrahepatic cholestasis (PFIC types 1 and 2). Diagnostic challenges were further compounded by co-existing factors, such as transiently elevated GGT levels due to CMV co-infection or concomitant medications (as seen in Case 6), which initially masked the classical BASD phenotype.

### Genetic findings

Cases 1 and 5 died prior to genetic testing. Three siblings from family 1 (Cases 2–4) were homozygous for a novel *HSD3B7* frameshift deletion, c.748_760del (p.Thr250Alafs*66), which is predicted to cause a loss of 3β-HSD function; both parents were heterozygous carriers. Case 6 (Family 3) was homozygous for the *AKR1D1* missense variant c.242A>T (p.Asp81Val). This variant results in a non-conservative amino acid substitution within the NADP-dependent oxidoreductase domain (IPR023210). It is predicted to be damaging by five independent *in silico* tools and carries an extremely low allele frequency in control populations (4.3 times 10^−6^). Although experimental functional evidence is currently lacking, this variant has been reported in multiple homozygous individuals with congenital BASD type 2 (21–23) and is registered in ClinVar (Variation ID: 499336).

Overall, four patients were considered to have 3β-HSD deficiency (Cases 1–4), with Case 1 presumed to be affected based on the familial diagnosis, one patient had 5β-R deficiency (Case 6), and one patient (Case 5) had an undetermined bile acid synthesis disorder.

### Treatment and outcomes

Three patients (Cases 3, 4, 6) were treated with CA and remain alive (Table 1). Initial doses of CA (6.2–8.4 mg/kg/day) were titrated upward, reaching maintenance doses of 10.7–13.4 mg/kg/day.

Cases 3 and 4, siblings diagnosed via family screening, started CA therapy at age 4 years (one month post- diagnosis). Hepatomegaly and steatorrhea rapidly resolved, with LFTs normalizing within 3–6 months. After 6 years, they remain asymptomatic, with normal growth, normal LFTs and no adverse events; liver elastography at age 10 years confirmed no fibrosis (F0). Their CA doses ranged from 8.3 to 11 mg/Kg/day. Persistently low levels of abnormal metabolites on urinary.

BAs analyses suggested either suboptimal dosing or imperfect adherence. This transient low dosing reflected a cautious stepwise titration, patient weight gain and limited drug availability, while adherence barriers (family psychological burden or temporary supply shortages) were managed with parental counseling and closer monitoring.

Case 6 started CA therapy at 18 months (6.2 mg/kg/day), achieving resolution of jaundice and steatorrhea within 2 months, and LFTs normalization by 6 months. After five years, the patient exhibits sustained clinical and biochemical remission. Based on an initial insufficient biochemical response, the dose was progressively up-titrated as planned, showing optimal reduction of abnormal metabolites at doses >10 mg/kg/day to reach a maintenance dose of 13.4 mg/kg/day.

Conversely, Cases 2 and 5 received UDCA (600 mg/m^2^/day) as an interim empirical therapy prior to definitive BASD confirmation. Both presented with ESLF at treatment initiation and died within 2–6 months. Liver transplantation could not be performed before death for Cases 1 and 2. All three deceased patients were either undiagnosed (Case 1) or lacked access to CA therapy (Cases 2 and 5). Death occurred at 5.5 years, 13.5 years, and 4 months, respectively, due to ESLF.

During the median follow-up of 6 years (range: 5–6 years for treated patients), CA treatment was well tolerated; no patients experienced dose-limiting side effects. Treatment adherence, monitored via parental interview, showed transient compliance drops occurring solely during temporary drug supply bottlenecks.

## Discussion

To the best of our knowledge, this is the first published case series of BASDs from Tunisia and North Africa. Our findings provide insights into phenotypic spectrum, genetic landscape and outcomes of BASDs in a previously under-reported population.

This study highlights the challenges of diagnosis, partly due to the heterogeneity of BASDs, as well as the difficulties of treatment access in resource-limited settings. It also confirms the efficacy and safety of CA therapy, consistent with previous reports (10).

### Epidemiological insights

While the exact global prevalence of BASDs remains unknown, estimated at a minimum of 1.13 cases per 10 million in Europe (8), incidence rates reach up to 2.7% in Saudi Arabia (13) and 6.3% in Japan (21) among patients with cholestasis or hepatic failure. In Tunisia, despite probable underdiagnosis, the estimated prevalence is higher (≈ 6.5 cases per 10 million). Geographic clustering in northwestern Tunisia and documented consanguinity in our cohort align with both the autosomal recessive nature of BASDs and the high consanguinity rates (15.4%–100%) reported in the literature. The observed male-only predominance (6/6) is likely coincidental rather than a specific genetic feature, though ascertainment bias remains possible.

### Diagnostic challenges

The age of onset in our series ranged from neonatal period to 8 years. Classically, 5β-R deficiency presents earlier (3 days–5 months) than 3β-HSD deficiency (8 weeks–3 years), due to a more toxic metabolite accumulation, although neonatal and late onset cases (up to the fourth decade) are not uncommon (5, 7).

These findings perfectly illustrate the marked phenotypic heterogeneity of BASDs, characterized in our cohort by three overlapping presentations:

- *Cholestatic phenotype* is the most recognized presentation. Typically presenting with normal or low GGT, this phenotype was characterized in cases 2, 5 and 6 by absent pruritus and paradoxically low serum BAs despite significant cholestasis, key clues differentiation BASDs from progressive familial intrahepatic cholestasis types 1 and 2 (22). Crucially, an elevated GGT level should not rule out BASDs, as it can be secondary to concurrent UDCA therapy, infections, or drugs interactions, as observed in Case 6.
- *Malabsorptive phenotype* illustrated by Cases 1–4, is an increasily recongnized presentation involving steatorrhea and features of FSV deficiencies (areflexia, hemorrhage, rickets) (13, 23). Elevated Creatine Kinase (Cases 1 and 2), likely reflects musculoskeletal dysfunction secondary to vitamin E and/or D deficiency.
- *Non-cholestatic hepatopathy* illustrated by Case 1 who presented at 8 years of age, is an atypical presentation that often delays diagnosis. Similar cases underscore that BASDs should be considered in any child with unexplained chronic hepatopathy, even without cholestasis (12, 13).

The intra-familial variability observed in Family 1 aligns with other series (13, 21, 24), suggesting that epigenetics, environmental or drugs exposures, the gut microbiome composition, or genetic modifiers influence disease progression (25).

Diagnostic reasoning in BASDs relies heavily on recognizing key biochemical discrepancies, particularly normal/low GGT cholestasis coupled with abnormally low serum total bile acids.

Diagnostic delays in our series, highlight the challenges of BASD rarity and phenotypic heterogeneity, exacerbated by limited access to specialized testing. Gold standard urinary BAs analyses by mass spectrometry (5), is unavailable locally, requiring costly and logistically complex sample shipment abroad. Consequently, genetic testing, despite being expensive and time-consuming for novel variants, is crucial for resolving atypical cases or false negative urinary BAs profiles (e.g., spontaneous remission, prior BAs therapy, technical errors, or early neonatal sampling) (7, 26).

The novel *HSD3B7* variant (c.748_760del), found in three siblings, expands the mutational spectrum of 3β-HSD deficiency, supported by disease segregation and absence in control databases. Meanwhile, the *AKR1D1* variant c.242A>T (p.Asp81Val) in Case 6 is a known mutation reported in Arab and Caucasian population (27), suggesting recurrence in the Mediterranean. Affecting a highly conserved residue in the 5β-R catalytic domain, this variant exhibits damaging *in silico* predictions and an extremely low control allele frequency. It has been documented in multiple homozygous individuals and is classified as pathogenic/likely pathogenic in ClinVar (ID: 499336) (28).

Prognosis in BASD is strictly governed by enzyme defect severity and diagnostic delay. Severe forms, such as 5β-R deficiency (Case 6), manifest early in infancy with rapid progression toward acute liver failure, whereas 3β-HSD deficiency can follow a more indolent course. Rapid molecular confirmation or family screening is paramount to establish prognosis and initiate lifesaving CA therapy prior to irreversible liver failure.

Early mortality precluded genetic subtyping for Case 5. However, the patient's rapidly progressive neonatal cholestasis and early liver failure strongly suggest a severe 5β-R deficiency phenotype, despite the absence of a documented familial link to Case 6.

### Cholic acid therapy: efficacy and access issues

Our findings outlined a stark contrast in outcomes, with mortality confined to untreated patients, while all CA treated patients survived. Nonetheless, baseline severity, age at diagnosis and the timing and duration of therapy remain critical prognostic determinants.

Importantly, while UDCA may support initial BASDs management by stimulating bile secretion, it remains inadequate for long-term therapy because it fails to suppress the production of endogenous hepatotoxic metabolites (5, 7, 27).

Conversely, CA therapy replaces missing primary BAs, restores bile flow, enables lipids and FSV absorption and suppresses hepatotoxic intermediates via negative feedback (7, 29). However, advanced liver failure at treatment initiation can limit its efficacy. In our cohort, all treated patients with CA achieved rapid and sustained remission. After 6 years, both patients with 3βHSD deficiency showed no liver fibrosis. These findings match long-term European data indicating a slowly progressive course in milder or late-diagnosed phenotypes (7, 12).

Lifelong CA therapy 10–15 mg/kg/day with regular follow-up are required (7, 29). The persistent abnormal urinary metabolites in Cases 3 and 4 highlight the necessity of strict biochemical monitoring and weight-based dose titration. The excellent 6-year safety profile in our patients aligns with literature reports showing that rare adverse events (diarrhea, pruritus, dehydration, transient increases in GGT, ALT, and BAs in serum) are typically dose-dependent (7, 10, 12, 27).

In Tunisia, access to CA was hindered by administrative and financial barriers, delaying therapy for Cases 2 and 5, who died before CA therapy could initiated. These fatal outcomes highlight the critical need for improved orphan drug availability in developing nations.

Given the poor natural prognosis of untreated BASDs, prompt initiation of therapy is paramount. Although cholic acid therapy represents a significant therapeutic investment, early treatment has the potential to substantially reduce disease progression, delay or prevent the need for liver transplantation, and lessen the burden of long-term complications. Compared with liver transplantation-which entails markedly higher healthcare expenditures, major surgical risks, and lifelong management with immunosuppressive therapy-early medical treatment may provide considerable clinical and healthcare benefits. Ensuring timely diagnosis and uninterrupted access to orphan drug therapy is therefore essential to optimize patient outcomes and reduce the overall burden of disease.

### Strengths and study limitations

The primary strength of this study lies in providing the first long-term clinical, biochemical, and molecular characterization of BASD in North Africa, introducing a novel pathogenic *HSD3B7* variant to the global mutational database. However, several limitations must be acknowledged.

First, the retrospective design and single-center tertiary setting introduce potential selection and referral bias, favoring more severe or classic presentations.

Second, early patient mortality (Cases 1, 2, and 5) precluded complete molecular and urinary bile acid subtyping in the entire cohort.

Third, while our maximum follow-up of 6 years is shorter than larger published cohorts (up to 24 years) (12), limited local availability of serum BAs and tandem mass spectrometry constrained our ability to perform a comprehensive monitoring of the outcome. Nevertheless, participation in a diagnostic support program successfully improved urinary BAs tracking and optimized CA titration.

Furthermore, an important limitation of our study is that genetic analysis was restricted to the two most common bile acid synthesis defects (HSD3B7 and AKR1D1). Consequently, patients harboring rarer genetic defects in the pathway may not have been identified, which could lead to an underestimation of the true genetic spectrum and prevalence of BASDs in our study population.

### Implications for practice

Our findings highlight the need for a greater awareness of atypical BASDs presentations and key diagnosis clues, early initiation and optimal dosing of CA therapy, and systematic family counseling and screening. Siblings should be screened, regardless of age or symptoms (6), as early treatment can prevent disease progression (as in cases 3 and 4). Prenatal diagnosis remains debated given the treatable nature of BASDs.

## Patient and caregiver perspective

Qualitative feedback obtained from the parents during routine clinical follow-ups revealed the profound emotional and psychosocial impact of BASDs. Families described years of diagnostic uncertainty and anxiety while seeking medical answers across multiple health centers. The initiation of CA therapy marked a dramatic turning point, bringing immediate psychological relief as clinical symptoms rapidly resolved. However, caregivers highlighted the ongoing stress associated with orphan drug access in Tunisia, including supply uncertainties, complex administrative procedures, and the burden of ensuring strict daily adherence to lifelong treatment.

## Conclusion

This first Tunisian series illustrates phenotypic heterogeneity and likely underdiagnosis of BASDs in Tunisia and North Africa, due to limited awareness and restricted diagnostic access. Identification of a novel *HSD3B7* variant expands the mutational spectrum of 3β-HSD deficiency.

Improved recognition, easier access to diagnostic tests, earlier and optimized CA therapy are essential to prevent disease progression and ensure normal survival.

## Data Availability

The raw data supporting the conclusions of this article will be made available by the authors, without undue reservation.
